# Supplementary material for: Synchronized long-read genome, methylome, epigenome, and transcriptome for resolving a Mendelian condition
Source: bioRxiv. 2023 Sep 27:2023.09.26.559521. Preprint. [Version 1] doi: 10.1101/2023.09.26.559521 (PMC10557686; doi:10.1101/2023.09.26.559521)
Supplement: Supplement 1 [file NIHPP2023.09.26.559521v1-supplement-1.pdf]

bioRxiv preprint doi: https://doi.org/10.1101/2023.09.26.559521; this version posted September 27, 2023. The copyright holder for this preprint (which was not certified by peer review) is the author/funder, who has granted bioRxiv a license to display the preprint in perpetuity. It is made available under aCC-BY 4.0 International license.

Supplementary Table 1. Clinical evaluation of proband

Biochemical test	Result
MMA	2.00 mcmol/L (Reference range <0.30) High
Homocysteine	5.6 umol/L (Reference range <9) Normal
Vitamin B12	1,581 pg/mL (Reference range 213-816) High
Plasma Amino Acids	Normal with Alanine 464 (Reference range 145-495)
Very long chain fatty acids (VLCFA)	Normal
Urine Organic Acids	Increased excretion of methylmalonic acid and trace amounts of methylcitric acid.
CK	Normal
GDF15	Elevated
Blood Lactate	5.9 mmol/L High
Ammonia	42 umol/L Normal
Plasma CMMPP	Cobalamin, methionine, and MMA pathways; not suggestive of disorder of MMA metabolism
ETC Enzyme analysis (muscle)	Normal

Imaging test	Result
Echocardiogram	Patent Foramen Ovale (PFO). Otherwise normal
MRI Brain	1. Suggestion of polymicrogyria predominating within the bilateral frontal lobes. It is noted that a syndrome of frontal predominant polymicrogyria, bilateral retinoblastoma, hypotonia, and dysmorphic facies has been described in the setting of an interstitial 13q deletion. Correlation with genetic testing is suggested.<br>2. No abnormal intracranial enhancement or evidence of pituitary or pineal mass.<br>3. Foci of susceptibility artifact along the bilateral caudothalamic grooves suggests sequela of remote hemorrhage.<br>4. No acute intracranial hemorrhage or infarct.
MRI Orbit	1. Enhancing bilateral ocular masses consistent with bilateral retinoblastomas confined to the globes. These measure up to 0.4 x 1.2 x 0.8 cm on the right and 0.3 x 0.8 x 0.6 cm on the left.

bioRxiv preprint doi: https://doi.org/10.1101/2023.09.26.559521; this version posted September 27, 2023. The copyright holder for this preprint (which was not certified by peer review) is the author/funder, who has granted bioRxiv a license to display the preprint in perpetuity. It is made available under aCC-BY 4.0 International license.

**Pathology test**	**Result**
Right thigh muscle biopsy	Electron microscopy examination of the submitted previously frozen muscle biopsy reveals the fibers to display indistinct myofibrillary organization, favored to represent an artifact of specimen preservation. Deviation from normal ultrastructural morphology includes degenerate mitochondria appearing as electron dense membranous inclusions within the myocyte and endothelial cells. The mitochondria are otherwise normal in distribution and quantity, with most displaying no significant morphologic abnormalities. It is possible these prepresent the pathologic expression of a primary mitochondriopathy; however, artifactual degeneration of the mitochondria resulting from freezing of the tissue and delayed fixation cannot be excluded. No nemaline bodies or other unusual structures are detected. Reduplications of the basement membrane are not seen. No tubuloreticular inclusions are observed. No unusual lysosomal storage product is found. Glycogen is present in normal amount. Lipid is not increased.

**Retinoblastoma history**	
Initial diagnosis:	5 months
Presenting Stage:	Right Eye: cT2a (C), Left Eye: cT1b (B)
Treatment:	Chemotherapy + Laser therapy + cryotherapy

**Physical exam**
High arched palate
Retrognathia
Hypotonia
Wide Nasal bridge
Telecanthus
Widow's peak
Pierre Robin sequence

bioRxiv preprint doi: https://doi.org/10.1101/2023.09.26.559521; this version posted September 27, 2023. The copyright holder for this preprint (which was not certified by peer review) is the author/funder, who has granted bioRxiv a license to display the preprint in perpetuity. It is made available under aCC-BY 4.0 International license.

Supplementary Table 2. Summary of prior genetic evaluations for proband

**Genetic test**	**Result**	**Interpretation**
SNP array	arr(1-22,X)x2	Nondiagnostic.
PWS/AS methylation	Normal pattern	Nondiagnostic.
Comprehensive neuromuscular panel	Negative	Nondiagnostic.
Karyotype and FISH for *RB1*	46,XX,t(X;13)(p22.1;q14.1)	Apparently balanced X;13 translocation; RB1 is unlikely to be disrupted at the breakpoint.
Cellular energetics panel +mtDNA	m.14568C>T (p.Gly36Ser) Pathogenic variant at 4% heteroplasmy	Nondiagnostic. m.14568C>T (p.Gly36Ser) thought to be unrelated to phenotype d/t low heteroplasmy in both fibroblast and blood sample.
RB1 sequencing	Negative	Nondiagnostic.
RB1 RNA sequencing	No variants reported; RB1 expression "low end of normal"	Indeterminate.
Trio exome sequencing +CNVs and mtDNA	Heterozygous maternally inherited *ABCC6* c.1850C>G (p.Ser617Ter) Likely Pathogenic variant Heterozygous paternally inherited *LAMC2* c.508C>T (p.Arg170Ter) Likely Pathogenic variant	Nondiagnostic. Heterozygous variants in genes associated with recessive phenotypes that do not match patient's phenotype.
Trio genome sequencing +mtDNA	m.14568C>T (p.Gly36Ser) Pathogenic variant at 5% heteroplasmy Heterozygous maternally inherited ABCC6 c.1850C>G (p.Ser617Ter) Likely Pathogenic variant Heterozygous paternally inherited LAMC2 c.508C>T (p.Arg170Ter) Likely Pathogenic variant	Nondiagnostic. m.14568C>T (p.Gly36Ser) thought to be unrelated to phenotype d/t low heteroplasmy in both fibroblast and blood sample. Heterozygous variants in genes associated with recessive phenotypes that do not match patient's phenotype.
m.14568C>T Urine Testing	Negative	Nondiagnostic.
Maternal karyoptyes	46,XX	Normal
Paternal karyotype	46,XY	Normal
